# A scintigraphy study of budesonide/glycopyrrolate/formoterol fumarate metered dose inhaler in patients with moderate-to-very severe chronic obstructive pulmonary disease

**DOI:** 10.1186/s12931-021-01813-w

**Published:** 2021-10-07

**Authors:** Omar Usmani, Nicolas Roche, Ezanul Wahab, Samuel Israel, Martin Jenkins, Roopa Trivedi, Paul Dorinsky, Magnus Aurivillius

**Affiliations:** 1 Asthma Lab, National Heart and Lung Institute (NHLI), Imperial College London & Royal Brompton Hospital, South Block, Royal Brompton Campus, Sydney St, Chelsea, London, SW3 6NP UK; 2 Respiratory Medicine, Hôpital Cochin (AP-HP), University of Paris, Cochin Institute, Paris, France; 3 Simbec Research Ltd, Merthyr Tydfil, UK; 4 AstraZeneca, Cambridge, UK; 5 AstraZeneca, Durham, NC USA; 6 AstraZeneca, Gothenburg, Sweden

**Keywords:** Pulmonary deposition, Gamma scintigraphy, Budesonide, Glycopyrronium, Formoterol fumarate

## Abstract

**Background:**

Triple therapy with inhaled corticosteroids/long-acting muscarinic antagonists/long-acting β_2_-agonists (ICS/LAMA/LABA) is recommended for patients with chronic obstructive pulmonary disease (COPD) with continued symptoms or exacerbations, despite treatment with LAMA/LABA or ICS/LABA. The pulmonary, extrathoracic, and regional lung deposition patterns of a radiolabeled ICS/LAMA/LABA triple fixed-dose combination budesonide/glycopyrrolate/formoterol fumarate (BGF 320/18/9.6 μg), delivered via a single Aerosphere metered dose inhaler (MDI) were previously assessed in healthy volunteers and showed good deposition to the central and peripheral airways (whole lung deposition: 37.7%). Here, we report the findings assessing BGF in patients with moderate-to-very severe COPD.

**Methods:**

This phase I, single-dose, open-label gamma scintigraphy imaging study (NCT03906045) was conducted in patients with moderate-to-very severe COPD. Patients received two actuations of BGF MDI (160/9/4.8 μg per actuation) radiolabeled with technetium‑99‑pertechnetate, not exceeding 5 MBq per actuation. Immediately following each inhalation, patients performed a breath-hold of up to 10 s, then exhaled into an exhalation filter. Gamma scintigraphy imaging of the anterior and posterior views of the lungs and stomach, and a lateral head and neck view, were performed immediately after exhalation. The primary objective of the study was to assess the pulmonary deposition of BGF. Secondary objectives assessed the deposited dose of radiolabeled BGF in the oropharyngeal and stomach regions, on the actuator, and on the exhalation filter in addition to regional airway deposition patterns in the lungs.

**Results:**

The mean BGF emitted dose deposited in the lungs was 32.1% (standard deviation [SD] 15.6) in patients with moderate-to-very severe COPD, 35.2% (SD 12.8) in patients with moderate COPD, and 28.7% (SD 18.4) in patients with severe/very severe COPD. Overall, the mean normalized outer/inner ratio was 0.55 (SD 0.19), while the standardized central/peripheral ratio was 2.21 (SD 1.64).

**Conclusions:**

Radiolabeled BGF 320/18/9.6 μg was efficiently delivered and deposited throughout the entire lung, including large and small airways, in patients with moderate-to-very severe COPD, with similar deposition in patients with moderate COPD and patients with severe/very severe COPD.

*Trial registration:* ClinicalTrials.gov, NCT03906045. Registered 8 April 2019, https://clinicaltrials.gov/ct2/show/NCT03906045

## Introduction

Patients with chronic obstructive pulmonary disease (COPD) may be prescribed treatment with triple therapy (inhaled corticosteroid/long-acting muscarinic antagonist/long-acting β_2_-agonist [ICS/LAMA/LABA]) if they continue to experience symptoms or exacerbations with dual therapies (LAMA/LABA or ICS/LABA) [1].

Ideally, an inhaled treatment would be consistently deposited in all parts of the lung, including the peripheral airways [2]. Gamma scintigraphy permits the assessment of these regional lung deposition patterns [3–7]. There can be extensive variation in both overall lung deposition and regional deposition patterns of inhaled therapies, dependent on multiple factors, such as the inhalation pattern and delivery device (e.g. metered dose inhaler [MDI] and dry powder inhaler [DPI]), as well as between different devices of the same type. Lung deposition with MDIs may be influenced by the mass of the drug, where a greater mass may reduce the percentage deposited in the lung, in addition to other factors, including the metered volume size and the propellant vapor pressure. Therefore, each MDI product may have unique lung deposition characteristics [8].

The ICS/LAMA/LABA budesonide/glycopyrrolate/formoterol fumarate (BGF), delivered twice daily via an Aerosphere inhaler (an MDI), is a triple fixed-dose combination therapy that has been approved for the maintenance treatment of COPD in the US, EU, and China, as well as in Japan, to relieve symptoms of COPD [9–12]. BGF was formulated as a suspension with micronized budesonide, micronized glycopyrronium bromide, and micronized formoterol fumarate crystals that were co-suspended with spray-dried porous particles in a hydrofluoroalkane (HFA) propellant (i.e. a single Aerosphere inhaler). Co-suspension delivery technology in the Aerosphere inhaler results in a strong, non-specific association between micronized drug crystals and phospholipid-based porous particles when they are suspended together in the MDI propellant HFA 134a (1,1,1,2-tetrafluoroethane). The resulting suspension is therefore uniform, with consistent dose delivery, even in the presence of simulated patient handling errors, such as a delay between shaking and actuation [13]. The ETHOS study (NCT02465567), which utilized devices with co-suspension delivery technology, showed that BGF significantly reduced moderate or severe COPD exacerbations, and improved lung function and symptoms, compared with corresponding ICS/LABA and LAMA/LABA therapies [14–16]. In addition, significant reductions in risk for all-cause mortality with BGF, compared with the LAMA/LABA glycopyrrolate/formoterol fumarate, were seen in patients with moderate-to-very severe COPD (unadjusted p = 0.0035) [17].

A previous gamma scintigraphy phase I study evaluated the lung deposition of radiolabeled BGF in healthy male volunteers (NCT03740373) and showed BGF was efficiently deposited in the central and peripheral regions of the lungs, following a 10- and 3-s breath-hold with an emitted dose in the lungs of 37.7% and 34.5%, respectively [6]. This study assessed the pulmonary deposition data for radiolabeled BGF, delivered via a single Aerosphere inhaler, in patients with moderate-to-very severe COPD.

## Methods

### Study design and treatment

**Fig. 1:**
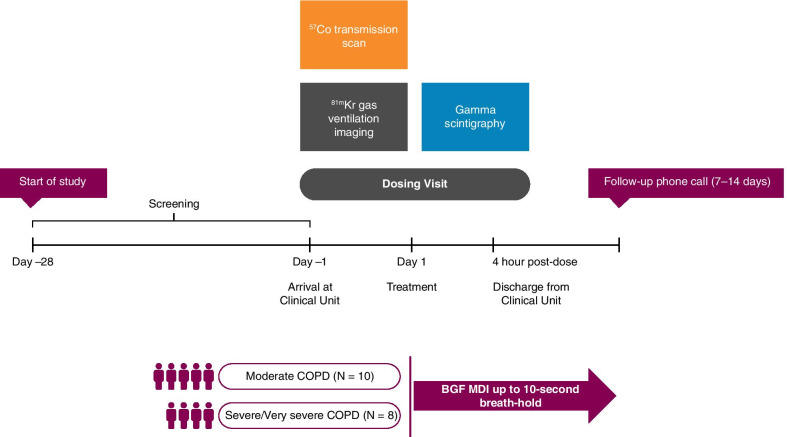
Study design. ^*57*^*Co* cobalt-57, ^*81m*^*Kr* Krypton-81 m, *BGF* budesonide/glycopyrrolate/formoterol fumarate, *COPD* chronic obstructive pulmonary disease, *MDI* metered dose inhaler

This was a phase I, single-dose, open-label gamma scintigraphy imaging study (NCT03906045) in patients with moderate-to-very severe COPD (Fig. 1). The screening visit was within 28 days of commencement of the dosing visit and included inhaler training (see below). During the treatment period, patients underwent a Krypton-81 m (^81m^Kr) gas ventilation scan to define the ventilated area of the lungs. A Cobalt-57 transmission scan was then performed to evaluate the regional tissue attenuation of deposited radioactivity. BGF was radiolabeled with technetium‑99‑pertechnetate (no greater than 5 MBq per actuation).

Eligible patients received a single dose of radiolabeled BGF 320/18/9.6 µg, consisting of two puffs of 160/9/4.8 μg per actuation, via a single Aerosphere inhaler. There was a breath-hold of up to 10 s after each inhalation. Gamma scintigraphy imaging was conducted immediately following completion of dosing procedures. Posterior and anterior views of the lungs and stomach, and a lateral head and neck view, were recorded using a gamma camera. Patients were discharged 4 h post-dose on Day 1, provided there were no safety concerns. A follow-up phone call was conducted 7–14 days after discharge.

### Patients

#### Inclusion and exclusion criteria

At screening and prior to dosing, patients were eligible for participation if they were 40–80 years of age, had a diagnosis of COPD, as defined by the American Thoracic Society/European Respiratory Society, with a post-bronchodilator forced expiratory volume in one second (FEV_1_)/forced vital capacity ratio < 0.70 and a post-bronchodilator FEV_1_ < 80% predicted, were receiving one or more inhaled maintenance therapies (including at least one LAMA or LABA) for management of their COPD for at least four weeks prior to screening, were a current or former smoker with a history of at least 10 pack-years, and were able to demonstrate proper MDI inhalation technique.

Key exclusion criteria were a current diagnosis of asthma, COPD due to an α_1_-antitrypsin deficiency, or any clinically significant respiratory disorder other than COPD. Patients who had experienced a moderate or severe COPD exacerbation within 6 weeks prior to dosing (where the end date of the exacerbation was the last day of treatment with systemic corticosteroids and/or antibiotics) were also excluded.

#### Inhaler training

At screening, patients were trained by site staff on the correct use of an MDI using a commercially available MDI training simulator (Vitalograph Aerosol Inhalation Monitor [AIM]™; Vitalograph, UK) and an HFA propellant-only MDI. The AIM™ device was used to confirm that the patient could use the MDI device and stay within the targeted inspiratory flow rate range for the MDI.

On Day –1 (when patients arrived at the clinical unit) and pre-dose on Day 1, patients used a non-radiolabeled propellant-only MDI to confirm they were using the MDI correctly. At any point during the study, site staff re-trained the patient if it was observed that the patient was not using the MDI correctly.

### Dose administration

Patients took two inhalations from the Aerosphere inhaler. Immediately following each inhalation, patients performed a breath-hold of up to 10 s, then exhaled into an exhalation filter. After the second exhalation and breath-hold, patients rinsed their mouth with approximately 20 mL of water and expelled the washings for collection. Patients then swallowed approximately a quarter of a slice of bread and approximately 100 mL of water to minimize interference from any residual radiolabeled particles in the mouth and throat on the gamma scintigraphy images. Patients continued their regular inhaled maintenance therapies for COPD through Day –1. On Day 1, regular maintenance therapies were withheld until discharge from the clinical unit and patients were provided ipratropium bromide hydrofluoroalkane (HFA) or albuterol sulfate HFA, which could be used up to, but not within, 6 h before BGF dosing.

### Gamma scintigraphy imaging

Gamma scintigraphy imaging was conducted immediately following completion of dosing procedures. Posterior and anterior views of the lungs and stomach, and a lateral head and neck view, were recorded using a gamma camera. Gamma scintigraphy images of the MDI were acquired before and after use. Additionally, images of the collected mouth washings and exhalation filter were acquired. All images were obtained over a maximum duration of 200 s.

Prior to the study, the radiolabeling procedures for BGF MDI were validated. In vitro tests were conducted to demonstrate that the aerodynamic particle size distribution of the radiolabel was the same as that for the micronized drug particles, as determined by in vitro Next Generation Impactor (NGI) tests (model 170, Copley Scientific Ltd., United Kingdom). The NGI is a cascade impactor that measures particle size (range of median diameters for model 170: 0.54 to 11.72 µm at 30 L per minute) by moving particles via an air stream that is forced through a series of 7 nozzles with progressively smaller diameters to produce successively higher velocities [18]. Radiolabeled MDIs showed no difference in performance relative to that of the non-radiolabeled control MDIs. On each dosing day prior to treatment administration, in vitro characterization of the radiolabeled MDI product was performed to ensure it complied with predefined release specifications.

### Study objectives and outcomes

The primary objective was to assess the pulmonary deposition of radiolabeled BGF in patients with moderate-to-very severe COPD following a maximal breath-hold of up to 10 s, with deposited doses in the lungs expressed as a percentage of the emitted dose.

Secondary objectives were to assess the deposited dose of radiolabeled BGF in the oropharyngeal and stomach regions, and on the actuator and exhalation filter, in addition to the regional airway deposition patterns in patients with moderate-to-very severe COPD following a maximal breath-hold of up to 10 s.

The deposited doses in the oropharyngeal and stomach regions, and on the exhalation filter, were expressed as a percentage of the emitted dose, and deposition on the actuator was expressed as a percentage of the ex-valve (metered) dose. Regional deposition ratios included outer/inner (O/I) and central/peripheral (C/P), penetration index (PI; equivalent to the normalized O/I) [19], and standardized C/P (sC/P) ratios [5]. These were calculated for the geometric mean image of the right lung only, as stomach-associated activity may interfere with the left lung image. Regional lung volumes were normalized by comparison to the ^81m^Kr gas ventilation scan in each patient, allowing for the definition of a rectangle and region of interest to define outer, inner, central, and peripheral areas. O/I and C/P counts were then obtained for the ^81m^Kr gas ventilation scan and these values used to account for differences in regional lung volumes. PI represents the regional distribution of the aerosol particles normalized for regional lung volume, and sC/P represents an alternative representation of the regional distribution of the aerosol particles normalized for regional lung volume using the ^81m^Kr gas ventilation scan.

The safety of radiolabeled BGF was assessed based on adverse events (AEs). All AEs were listed and coded using the Medical Dictionary for Regulatory Activities (MedDRA), version 22.0. Serious AEs were recorded from the time of consent to the follow-up phone call, and non-serious AEs were recorded from BGF dosing to follow-up phone call. Only treatment emergent AEs (TEAE) were to be summarized by cohort and overall (both cohorts combined).

### Statistical analysis

Patients were separated into two cohorts, moderate COPD and severe/very severe COPD, to permit an exploratory comparison. Since this was an exploratory comparison, no formal sample size calculation was performed; however, a sample size of at least 16 (eight/cohort) allowed a direct estimate of lung deposition from this formulation to be made, which is similar to other investigational scintigraphy studies [20, 21].

The primary analysis consisted of descriptive statistics for the primary and secondary endpoints conducted in the per protocol (PP) analysis set, which comprised all patients who received a dose of BGF, had fully evaluable scintigraphy data, and had no protocol violations that may have invalidated or biased the results. A supportive analysis was conducted in patients who received any amount of BGF (safety analysis set). Descriptive statistics were produced for the overall analysis set and within each severity cohort.

Deposition of BGF in the lungs of patient cohorts with moderate vs. severe/very severe COPD was also assessed. Data in these cohorts were analyzed using an analysis of covariance (ANCOVA) that included COPD severity as a fixed effect, sex as a cofactor, and age, height, and breath-hold duration as covariates. Differences in deposition between moderate COPD and severe/very severe COPD were reported as least squares mean (LSM) (± standard error [SE]), along with point estimates and 95% confidence intervals (CIs).

## Results

### Radiolabeling validation

**Fig. 2:**
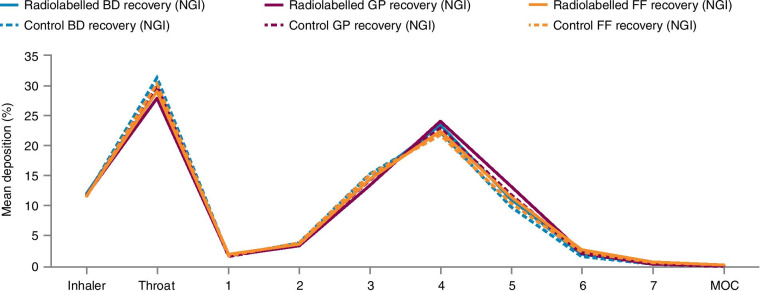
Aerodynamic particle size distribution from BGF MDIs. Comparison of budesonide, glycopyrrolate, and formoterol fumarate aerodynamic particle size distribution from non-radiolabeled BGF MDIs and radiolabeled BGF MDIs used in this study. The effective cut-off particle size for each stage is shown for an inhalation flow rate of 30 L/min. *BD* budesonide, *BGF* budesonide/glycopyrrolate/formoterol fumarate, *FF* formoterol fumarate, *GP* glycopyrrolate, *MOC* micro-orifice collector, *MDI* metered dose inhaler, *NGI* Next Generation Impactor

The NGI analytical tests conducted during radiolabeling method development demonstrated that the aerodynamic size distribution of the emitted aerosol particles for each of the analytes, i.e. budesonide, glycopyrronium, and formoterol fumarate, were comparable to those from the non-radiolabeled canisters, confirming that the radiolabeling process did not alter the performance relative to that of the control non-radiolabeled MDIs. The deposition patterns of each of the analytes are presented in Fig. 2. The effective cut-off diameter (μm) for each stage is shown for a flow rate of 30 L/min, as is the Pharmacopoeial standard [22]. The fine particle fraction for radiolabeled budesonide ranged from 48.69–56.48% (vs. 46.40–53.51% for unlabeled), for glycopyrronium ranged from 51.43–59.45% (vs. 48.74–56.49% for unlabeled), and for formoterol fumarate ranged from 49.31–56.76% (vs. 48.03–54.59% for unlabeled). Tests immediately prior to administration of radiolabeled aerosol on the dosing day also confirmed that each radiolabeled canister delivered the correct amount of radioactivity. Validation of the radiolabeling process confirmed that the radiolabeled aerosols fulfilled criteria described by Devadason and colleagues [3].

### Patients

**Fig. 3:**
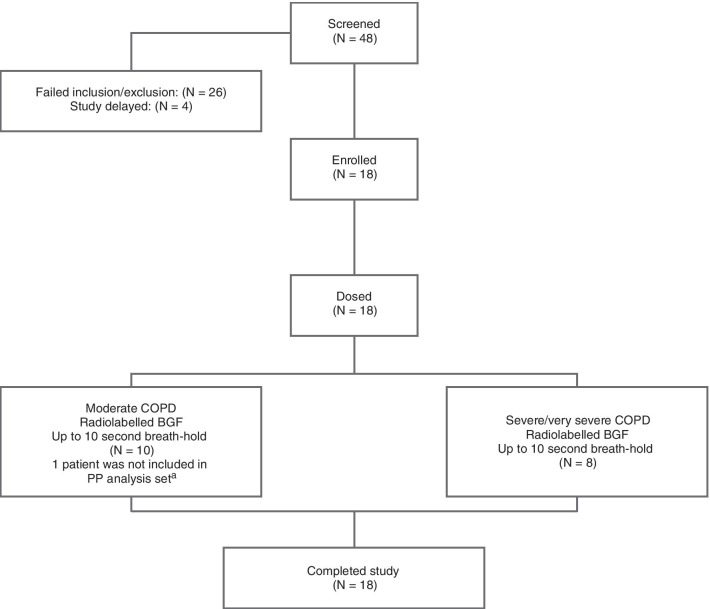
Patient disposition. ^a^One patient was excluded due to improper MDI inhalation technique. *BGF* budesonide/glycopyrrolate/formoterol fumarate, *COPD* chronic obstructive pulmonary disease, *MDI* metered dose inhaler, *PP* per protocol

Of the 48 patients screened, 18 patients were enrolled and dosed (moderate COPD, N = 10; severe/very severe COPD, N = 8; Fig. 3). Seventeen patients were included in the PP analysis set, since one patient with moderate COPD was excluded due to improper MDI inhalation technique. All patients (N = 18) were included in the safety analysis set.

All enrolled patients were white, 55.6% were male, with an overall mean age of 62.0 years, and mean FEV_1_ of 51.1% predicted. Mean breath-hold time was 9.6 s (Table 1), with breath-hold duration comparable for patients with moderate and severe/very severe COPD. Of the 8 patients in the severe/very severe COPD cohort, 7 had severe COPD and 1 had very severe COPD.

**Table 1:** Summary of patient demographics and baseline characteristics (safety analysis set)

Parameter	Moderate COPD (N = 10)	Severe/very severe COPD (N = 8)	Overall (N = 18)
Mean age in years (SD)	61.3 (10.8)	62.9 (5.7)	62.0 (8.7)
Male, n (%)	6 (60.0)	4 (50.0)	10 (55.6)
White, n (%)	10 (100)	8 (100)	18 (100)
Mean BMI, kg/m^2^ (SD)	27.0 (3.2)	26.5 (4.2)	26.8 (3.6)
Mean years since COPD diagnosis (SD)	6.6 (6.7)	7.8 (6.3)	7.1 (6.4)
Mean breath-hold duration, seconds (SD)	9.5 (1.0)	9.7 (0.7)	9.6 (0.8)
Mean FEV_1_% predicted (SD)	61.1 (8.7)	38.6 (7.4)	51.1 (13.9)

*BMI* body mass index, *COPD* chronic obstructive pulmonary disease, *FEV*_*1*_ forced expiratory volume in one second, *SD* standard deviation

### Lung deposition

**Fig. 4:**
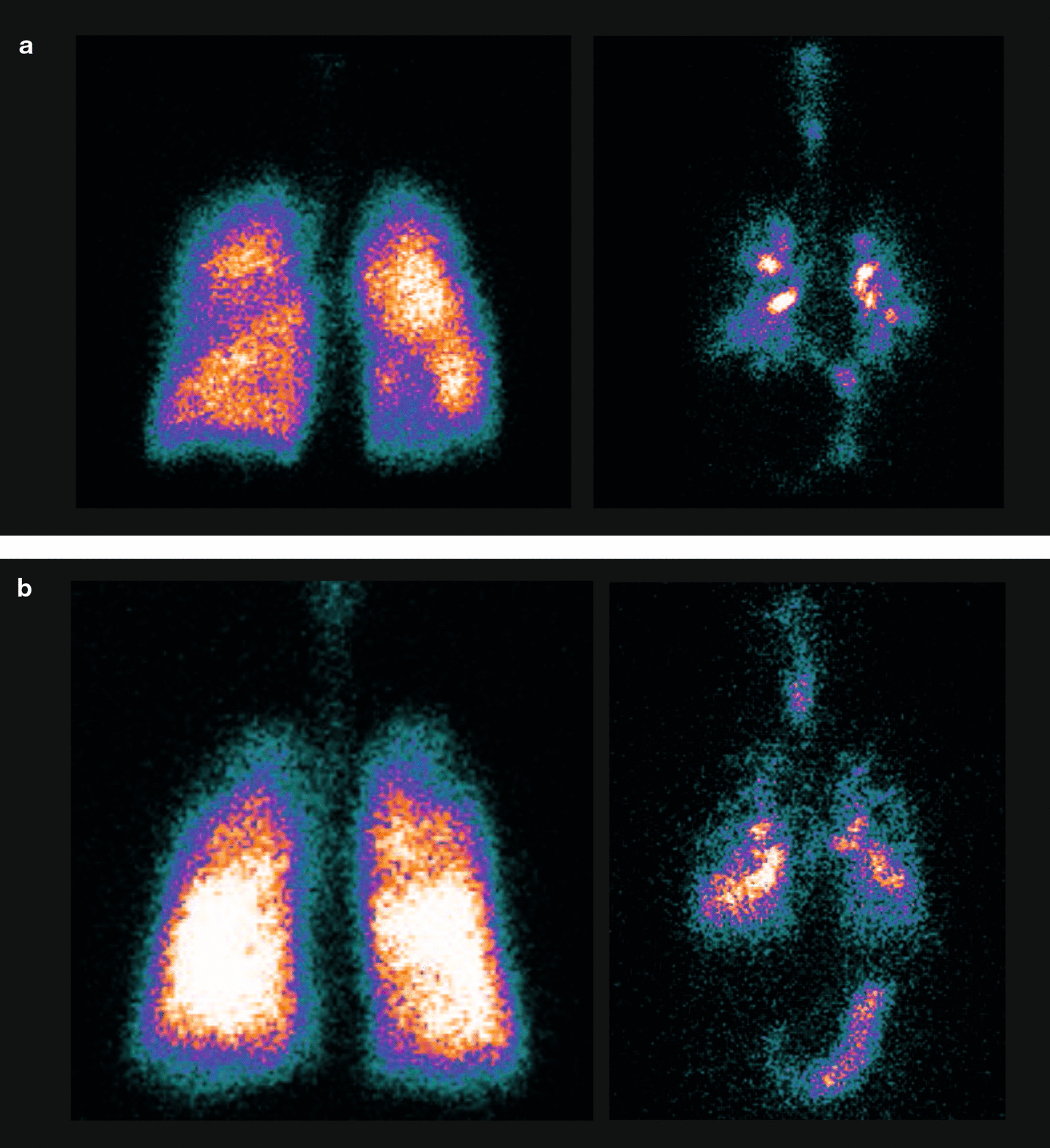
Representative gamma scintigraphy posterior view images of ^81m^Kr gas ventilation (left) and lung deposition of BGF (right) in a patient with **a** moderate COPD and a patient with **b** severe/very severe COPD (PP analysis set). ^*81m*^*Kr* Krypton-81 m, *BGF* budesonide/glycopyrrolate/formoterol fumarate, *COPD* chronic obstructive pulmonary disease, *PP* per protocol

Gamma scintigraphy images for the ^81m^Kr gas ventilation scan and BGF deposition in representative patients from each cohort are shown in Fig. 4.

**Fig. 5:**
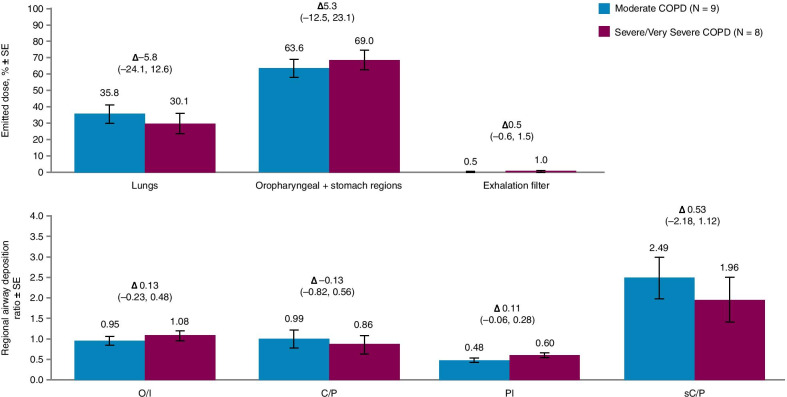
Derived deposition data for percentage emitted dose and regional deposition ratios in the lung by COPD severity (PP analysis set). *∆* LSM difference for severe/very severe vs. moderate COPD (95% CI), *CI* confidence interval, *COPD* chronic obstructive pulmonary disease, *C/P* central to peripheral, *LSM* least squares mean, *O/I* outer to inner, *PI* penetration index, *PP* per protocol, *sC/P* standardized central/peripheral, *SE* standard error

The mean percentage of the BGF emitted dose deposited in the lungs was 32.1% in the overall PP analysis set; 35.2% for patients with moderate COPD and 28.7% for patients with severe/very severe COPD (Table 2; Fig. 5). The estimated difference in BGF emitted dose deposited in the lungs in patients with severe/very severe COPD relative to patients with moderate COPD was minimal (Table 3).

**Table 2:** Summary of derived deposition data^a^ by COPD severity (PP analysis set)

Cohort	Variable	Mean	SD	Median	Min, Max
Overall (N = 17)^b^	Emitted dose in lungs, %	32.1	15.6	33.0	6.4, 52.1
	Emitted dose in oropharyngeal + stomach regions, %	67.2	15.1	66.2	47.5, 93.9
	Emitted dose in exhalation filter, %	0.7	0.9	0.5	0.1, 3.8
	Ex-valve dose on actuator, %	11.2	1.7	11.0	9.2, 15.2
	Regional airway deposition ratio:				
	O/I	1.04	0.40	1.03	0.34, 1.95
	C/P	0.90	0.69	0.71	0.43, 2.94
	PI	0.55	0.19	0.54	0.17, 0.89
	sC/P	2.21	1.64	1.67	1.13, 7.19
Moderate COPD (N = 9)	Emitted dose in lungs, %	35.2	12.8	41.1	9.3, 52.1
	Emitted dose in oropharyngeal + stomach regions, %	64.3	12.5	58.6	47.5, 89.2
	Emitted dose in exhalation filter, %	0.6	0.4	0.4	0.2, 1.5
	Ex-valve dose on actuator, %	12.0	1.9	11.7	9.9, 15.2
	Regional airway deposition ratio:				
	O/I	0.93	0.31	1.03	0.34, 1.46
	C/P	1.01	0.74	0.83	0.48, 2.94
	PI	0.48	0.16	0.49	0.17, 0.74
	sC/P	2.56	1.79	2.03	1.28, 7.19
Severe/very severe COPD (N = 8)	Emitted dose in lungs, %	28.7	18.4	25.5	6.4, 51.5
	Emitted dose in oropharyngeal + stomach regions, %	70.4	17.9	74.0	48.4, 92.9
	Emitted dose in exhalation filter, %	0.9	1.2	0.5	0.1, 3.8
	Ex-valve dose on actuator, %	10.4	1.1	10.2	9.2, 12.7
	Regional airway deposition ratio:				
	O/I	1.17	0.48	1.20	0.40, 1.95
	C/P	0.78	0.66	0.54	0.43, 2.37
	PI	0.63	0.20	0.68	0.27, 0.89
	sC/P	1.81	1.45	1.35	1.13, 5.38

^a^Following a breath-hold of up to 10 s after each inhalation

^b^One patient (with moderate COPD) was excluded due to improper MDI inhalation technique

*COPD* chronic obstructive pulmonary disease, *C/P* central to peripheral, *MDI* metered dose inhaler, *Min* minimum, *Max* maximum, *O/I* outer to inner, *PI* penetration index, *PP* per protocol, *sC/P* standardized central/peripheral, *SD* standard deviation

**Table 3:** Lung deposition and regional airway deposition^a^ by COPD severity (PP analysis set)

Variable	Moderate COPD	Severe/very severe COPD	Severe/very severe vs. moderate COPD	
	LSM (SE) (N = 9)	LSM (SE) (N = 8)	Difference in LSM (SE)	95% CI
Emitted dose in lungs, %	35.8 (5.7)	30.1 (6.1)	− 5.8 (8.3)	− 24.1, 12.6
Emitted dose in oropharyngeal + stomach regions, %	63.6 (5.5)	69.0 (5.9)	5.3 (8.1)	− 12.5, 23.1
Emitted dose in exhalation filter, %	0.5 (0.3)	1.0 (0.3)	0.5 (0.5)	− 0.6, 1.5
Ex-valve dose on actuator, %	12.2 (0.6)	10.4 (0.6)	− 1.8 (0.8)	− 3.6, 0.04
Regional airway deposition ratio:				
O/I	0.95 (0.11)	1.08 (0.12)	0.13 (0.16)	− 0.23, 0.48
C/P	0.99 (0.21)	0.86 (0.23)	− 0.13 (0.31)	− 0.82, 0.56
PI	0.48 (0.05)	0.60 (0.06)	0.11 (0.08)	− 0.06, 0.28
sC/P	2.49 (0.51)	1.96 (0.55)	− 0.53 (0.75)	− 2.18, 1.12

^a^Following a breath-hold of up to 10 s after each inhalation

*COPD* chronic obstructive pulmonary disease, *CI* confidence interval, *C/P* central to peripheral, *LSM* least squares mean, *O/I* outer to inner, *PI* penetration index, *PP* per protocol, *sC/P* standardized central/peripheral, *SE* standard error

The mean percentage of the BGF emitted dose deposited in the oropharyngeal and stomach regions was 67.2% in the overall population. A low percentage of the emitted dose was detected in the exhalation filter for radiolabeled BGF. The mean percentage of radiolabeled BGF detected in the actuator was 11.2% in the overall population; similar amounts were detected for patients with moderate and severe/very severe COPD (Table 2).

The mean PI regional airway deposition ratio was 0.55 in the overall population, 0.48 in patients with moderate COPD, and 0.63 in patients with severe/very severe COPD. The mean sC/P regional airway deposition ratio was 2.21 for the overall population, 2.56 in patients with moderate COPD, and 1.81 in patients with severe/very severe COPD (Table 2).

### Safety

No AEs were reported in this single-dose study.

## Discussion

In patients with moderate-to-very severe COPD, the radiolabeled BGF formulation was efficiently deposited in the lungs, with similar regional deposition patterns for patients with both moderate and severe/very severe COPD. The mean percentage emitted dose in the lungs of patients with moderate COPD was approximately 6% higher than in patients with severe/very severe COPD, with a corresponding decrease in the mean value deposited in the oropharyngeal and stomach regions (including radioactivity detected in the mouthwash and in the stomach). While the overall magnitude of this difference was small, the fact that some difference was observed is not unexpected given the severity of airway disease in this COPD patient population and the considerable variation in lung deposition values for patients with moderate COPD (9.3–52.1%) and patients with severe/very severe COPD (6.4–51.5%). There were no safety concerns identified in this study.

A previous phase I gamma scintigraphy imaging study found that breath-hold duration of 3 vs. 10 s provided comparable deposition of radiolabeled BGF in healthy male volunteers [6]. Although the study reported herein used a breath-hold of 10 s (as is generally recommended for MDIs [23]), the methodology for radiolabeling the MDI product, patient training and dose administration, and analysis of gamma scintigraphy images were the same for both studies and therefore allows comparison. The mean percentage of pulmonary deposition for BGF was similar for patients with moderate-to-very severe COPD (32.1% [SD 15.6]) and for phase I study healthy male volunteers (37.7% [SD 15.2] [6]). Relative to other MDIs, overall lung deposition with the Aerosphere formulation can be considered similar in healthy volunteers and both cohorts of COPD patients [2, 6, 7, 24]. However, in terms of deposition estimates, mean drug deposition declined as lung function worsened, with healthy male volunteers having the highest deposition (37.7%) [6], followed by patients with moderate COPD (35.2%), then patients with severe/very severe COPD (28.7%). Lung deposition was lower in patients with severe/very severe COPD than in patients with COPD of a lesser severity in this study. However, it should be noted that lung deposition with an MDI in patients with severe and very severe COPD who may also have limited inspiratory flow is likely to be greater than would be associated with DPI use. In this regard, an in silico study investigating the influence of inspiratory flow capability and device type on total lung deposition showed that in patients with moderate-to-very severe COPD, use of a DPI was associated with lower total deposition (14–27%) vs. BGF MDI (40–48%). More uniform deposition of individual drug components was also observed for MDI vs. DPI use in that study [25]. Since suitable inspiratory flow is a criterion for effective DPI use (the patient must inhale ‘hard and fast’) [26], for patients with suboptimal inspiratory flow the use of an MDI may be preferable.

As discussed, the overall lung deposition in this study was similar to that described for other HFA dual therapy pressurized MDIs [2, 7] despite the different formulations of other drugs. However, in another study that examined a suspension formulation of fluticasone propionate/salmeterol HFA in patients with asthma, lung deposition was considerably lower (16%) than we observed in our study of patients with COPD (32.1%) [27].

For both O/I and C/P, it is important to correct for regional lung volumes by comparison with an ^81m^Kr gas ventilation scan in each patient. A value close to 1 indicates that the delivered aerosol is deposited throughout the lung airways, equally between large and small airways. Values of ≥ 1 for PI or ≤ 1 for sC/P indicate a greater proportion in the peripheral airways; conversely, values < 1 for PI or > 1 for sC/P indicate a greater proportion in the larger airways. Our data show that administration of BGF resulted in distribution of aerosol particles to all regions of the lungs, and that BGF was delivered to the lungs of patients with COPD with similar efficiency to healthy volunteers (mean PI, 0.55 (SD 0.19) and 0.65 (SD 0.20), respectively) [6]. Moreover, while BGF was distributed in all regions of the lung in both patients with COPD and healthy volunteers, there was a tendency for a somewhat more central deposition in patients with COPD compared to healthy volunteers (mean sC/P, 2.21 (SD 1.64) vs. 1.79 (SD 0.79), respectively) [6].

The low number of patients was a potential limitation of this study, however, the size of the study was similar to that of previous gamma scintigraphy studies (N = 3–12) [2, 6, 7, 20, 21, 28–30]. Another limitation was the wide variation in the lung deposition values in both severity groups (overall: 6.4–52.1%), despite all patients being carefully trained in the use of an MDI with an AIM inhalation monitor, which is able to visually indicate correct flow rate, co-ordination of actuation and inhalation, and breath-hold. However, this wide variation in lung deposition was also seen in healthy volunteers (20.3–68.3%) [6]. Although the patient population included patients with severe/very severe airflow, MDI devices pose little or no resistance to airflow and are therefore suited for this population. It is possible that despite training, some patients had a suboptimal MDI inhalation technique. If this is the case, delivery of BGF can be improved with a spacer device [31].

## Conclusions

In conclusion, these results indicate that BGF 320/18/9.6 µg was efficiently deposited throughout the entire lung in patients with moderate-to-very severe COPD. Importantly, deposition was generally similar in patients with moderate COPD and patients with severe/very severe COPD. The results of this study in patients with COPD were generally comparable with BGF deposition observed in prior studies with healthy volunteers, suggesting that despite significant airway disease, BGF is deposited to the entire lung, including the large and small airways.

## Data Availability

Data underlying the findings described in this manuscript may be obtained in accordance with AstraZeneca’s data sharing policy described at https://astrazenecagrouptrials.pharmacm.com/ST/Submission/Disclosure.

## Abbreviations

AAD: Adaptive Aerosol Delivery
AAPS: American Association of Pharmaceutical Scientists
AE: Adverse event
AIM: Aerosol Inhalation Monitor
BGF: Budesonide/glycopyrrolate/formoterol fumarate
CI: Confidence interval
COPD: Chronic obstructive pulmonary disease
DPI: Dry powder inhaler
MDI: Metered dose inhaler
NGI: Next Generation Impactor
PI: Penetration index
PP: Per protocol
